# Completion of LINE integration involves an open ‘4-way’ branched DNA intermediate

**DOI:** 10.1093/nar/gkz673

**Published:** 2019-08-08

**Authors:** Brijesh B Khadgi, Aruna Govindaraju, Shawn M Christensen

**Affiliations:** Department of Biology, University of Texas at Arlington, Arlington, TX 76019, USA

## Abstract

Long Interspersed Elements (LINEs), also known as non-LTR retrotransposons, encode a multifunctional protein that reverse transcribes its mRNA into DNA at the site of insertion by target primed reverse transcription. The second half of the integration reaction remains very poorly understood. Second-strand DNA cleavage and second-strand DNA synthesis were investigated *in vitro* using purified components from a site-specific restriction-like endonuclease (RLE) bearing LINE. DNA structure was shown to be a critical component of second-strand DNA cleavage. A hitherto unknown and unexplored integration intermediate, an open ‘4-way’ DNA junction, was recognized by the element protein and cleaved in a Holliday junction resolvase-like reaction. Cleavage of the 4-way junction resulted in a natural primer-template pairing used for second-strand DNA synthesis. A new model for RLE LINE integration is presented.

## INTRODUCTION

Long interspersed elements (LINEs) are an abundant and diverse group of autonomous transposable elements (TEs) that are found in eukaryotic genomes across the tree of life. LINEs also mobilize the nonautonomous short interspersed elements (SINEs) which appropriate the protein machinery of LINEs to replicate. The movements of LINEs and SINEs have been implicated in genome evolution, modulation of gene expression, genome rearrangements, DNA repair, cancer progression, and as a source of new genes (1,2). LINEs replicate by a process called target primed reverse transcription (TPRT), where the element RNA is reverse transcribed into DNA at the site of insertion using a nick in the target DNA to prime reverse transcription (3–5). LINEs encode protein(s) that are used to perform the critical steps of the insertion reaction. LINE proteins bind their own mRNA, recognize target DNA, perform first-strand target–DNA cleavage and perform TPRT. The proteins are also hypothesized to perform second-strand target–DNA cleavage and second-strand element-DNA synthesis, although the evidence for these is sparse (3–20). The early-branching clades of LINEs encode a restriction-like endonuclease (RLE), while the later-branching LINEs encode an apurinic-apyrimidinic DNA endonuclease (APE) (21–24). Both types of elements are thought to integrate through a functionally equivalent integration process (5,25–27).

Second-strand DNA cleavage has remained unclear because the cleavage sites generally are not palindromic: the sequence around the second-strand cleavage site is often unrelated to the sequence around the first-strand cleavage site. In addition, blunt or staggered cleavages can occur. The staggered cleavages give rise to target site duplications or target site deletions depending on whether the staggered cut is 3′ overhanging or 5′ overhanging, respectively. Moreover, the staggered cleavages can be a few bases away (e.g. 2 bp in R2Bm) or quite distant (e.g. 126 bp in R9) (28,29). In APE LINEs, as in RLE LINEs, the cleavages are generally staggered such as to generate a modest 10–20 bp target site duplication upon insertion (26,30–32). The endonuclease from APE-bearing LINEs (APE LINEs) appears to have some specificity for the first DNA cleavage site, but much less so for the second DNA cleavage site on linear target DNA (23,30,31,33,34). The endonuclease from the RLE-bearing LINEs (RLE LINEs) is similarly involved in target site recognition (11). In both cases, however, additional specifiers for cleavage have been hypothesized to account for the different specificity of the first and second-strand DNA cleavages including the endonuclease being tethered to the DNA by unidentified DNA binding domains in the protein. Another complicating factor is that the first cleavage event should occur in the presence of element RNA, while the second cleavage event, according to *a priori* reasoning, should occur in the absence of element RNA, due to cDNA formation, however cleavage in the absence of RNA has been difficult to demonstrate *in vitro* (20).

Second-strand DNA synthesis has remained unresolved since TPRT was first described over 20 years ago, and it has never been directly observed *in vitro* (4,15,25,35,36). Second-strand synthesis (SSS) is hypothesized to be primed off the free 3′-OH generated by the second-strand cleavage event and synthesized by the element-encoded reverse transcriptase (RT). It is unknown how the proposed primer-template association is generated as the ends of the double-stranded cleaved target DNA drift away post cleavage in *in vitro* reactions (6,20).

**Figure 1.**
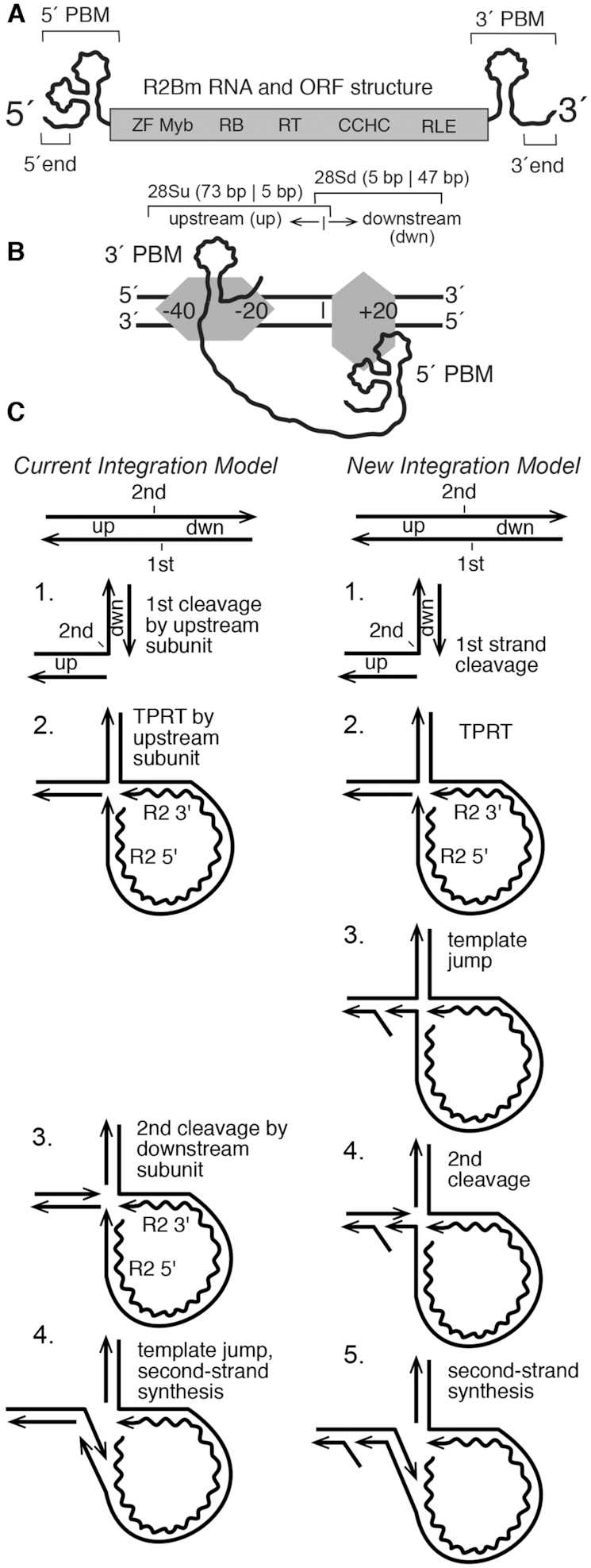
R2Bm structure and integration reaction. (**A**) R2Bm RNA (wavy line) and open reading frame (ORF) structure (gray box). The ORF encodes conserved domains of known and unknown functions: zinc finger (ZF), Myb (Myb), reverse transcriptase domain (RT), a cysteine-histidine rich motif (CCHC) and a PD-(D/E)XK type RLE. RNA structures present in the 5′ and 3′ untranslated regions that bind R2 protein are marked as 5′ and 3′ PBMs, respectively. The small (25 nt) RNA segments from the 5′ end and 3′ ends of the element RNA used in this study are indicated. (**B**) The R2 integration complex, as currently understood, is depicted bound to a segment of linear 28S rDNA (black parallel lines). An R2 protein subunit (gray horizontal hexagon) is bound upstream of the insertion site (vertical bar), and an R2 protein (gray vertical hexagon) subunit is bound downstream of the insertion site. The upstream subunit is associated with the 3′ PBM RNA, and the downstream subunit is associated with the 5′ PBM RNA. The footprints of the two protein subunits on the linear target DNA are indicated. The upstream subunit footprints from −40 bp to −20 bp, but it grows to just over the insertion site (vertical line) after first-strand DNA cleavage. The downstream subunit footprints from just prior to the insertion site to +20 bp (10,20). The overlapping portions of the target DNA, 28Su and 28Sd, used in this study are indicated with brackets. (**C**) The Current Integration Model and the New Integration Model being proposed in this paper are compared. Straight lines are DNA (28S or R2). The wavy line is the R2 RNA. The four steps of the current model are: (1) DNA cleavage of the bottom/first-strand of the target DNA; (2) TPRT; (3) DNA cleavage of the top/second strand of the target DNA; and (4) second-strand DNA synthesis. The fourth step has not been observed directly *in vitro*. The five steps of the new model are: (1) DNA cleavage of the bottom/first-strand of the target DNA; (2) TPRT; (3) a template jump/recombination event that generates an open ‘4-way’ DNA junction; (4) second-strand DNA cleavage; and (5) second-strand DNA synthesis. Abbreviations: up (target sequences upstream of the insertion site), dwn (target sequences downstream of the insertion site) and TPRT.

The R2 element from *Bombyx mori*, R2Bm, is one of a number of model systems that has been used to study the insertion reaction of LINEs (27). R2 elements are site specific, targeting the ‘R2 site’ in the 28S rRNA gene (27). The R2 element encodes a single open reading frame with N-terminal zinc finger(s) (ZF) and Myb domains (Myb), a central reverse transcriptase (RT), an RLE and a C-terminal gag-knuckle-like CCHC motif (Figure 1A). The R2Bm protein has been expressed in *Escherichia coli* and purified for use in *in vitro* reactions.

*In vitro* studies of the R2Bm protein and RNA have contributed to the current model of integration for R2Bm (Figure 1B and C) (20). Two subunits of R2 protein, one bound to the 3′ protein binding motif (PBM) of the R2 RNA and other to the 5′ PBM of the R2 RNA, are thought to be involved in the integration reaction. The 5′ and 3′ PBM RNAs dictate the roles of the two subunits and coordinate a series of DNA cleavage and polymerization steps, resulting in element integration by TPRT (Figure 1A). The protein subunit bound to the element's 3′ PBM interacts with 28S rDNA sequences upstream of the R2 insertion site. The upstream subunit's RLE cleaves the first (bottom/antisense) DNA strand. After first-strand target-DNA cleavage, the subunit's RT performs TPRT using the 3′-OH generated by the cleavage event to prime first-strand cDNA synthesis. The protein subunit bound to the 5′ PBM RNA interacts with 28S rDNA sequences downstream of the R2 insertion site by way of the ZF and Myb domains. The downstream subunit's RLE cleaves the second (top/sense) DNA strand, but only after the 5′ PBM RNA structure is destroyed by TPRT during cDNA formation, putting the protein in the minus RNA state. Second-strand DNA cleavage, however, is not thought to occur until after the 5′ PBM RNA is pulled from the subunit, presumably by the process of TPRT, putting the protein in a ‘no RNA bound’ conformation. Confusingly, second-strand DNA cleavage does not readily occur in the absence of RNA in our *in vitro* reactions. Second-strand cleavage had, until this report, required a narrow range of R2 protein, 5′ PBM RNA and target DNA ratios to be observed in *in vitro* reactions (20). Additionally, second-strand cleavage had, until this report, disconnected the primer for SSS, the 3′-OH generated by second-strand cleavage, from the cleavage event from the cDNA template, making initiation of second-strand DNA synthesis problematic (6,20).

The DNA endonuclease plays a central role in the integration reaction of LINEs. The RLE found in the early-branching LINEs is a variant of the PD-(D/E)XK superfamily of endonucleases (11,22). In a previous paper, we reported the similarity of the LINE RLE as having sequence and structural homology to archaeal Holliday junction resolvases (11,37). Our previous paper left open the question as to whether R2 protein could function on branched DNA molecules and what this potential activity tells us about the insertion reaction. Of particular interest is the TPRT product, a pseudo (i.e. open) ‘3-way’ junction, and a proposed open ‘4-way’ junction. The open 3- and 4-way junctions are key substrates that differentiate two models of insertion (Figure 1C): (i) The *Current Integration Model*, and (ii) a *New Integration Model* being proposed herein. The two models differ in the timing and substrate of second-strand DNA cleavage. Cleavage of the TPRT product (*Current Integration Model*) produces a fully cleaved target DNA with no obvious primer-template from which to prime second-strand DNA synthesis; it is proposed that a template jump occurs post DNA cleavage in order to prime SSS. In the *New Integration Model*, the proposed template jump/switch occurs prior to second-strand DNA cleavage and thus forms the 4-way-like junction. The open 4-way junction, upon DNA cleavage, resolves into a natural primer-template that could be used in second-strand DNA synthesis.

## MATERIALS AND METHODS

### Nucleic acid preparation and R2Bm protein purification

Oligonucleotides (oligos) containing 28S R2 target DNA, non-target (nonspecific) DNA and R2 sequences were ordered from Sigma-Aldrich. The upstream (28Su) and downstream (28Sd) target DNA designations are relative to the R2Bm insertion dyad within the 28S rRNA gene. The DNA constructs were formed by annealing the component oligos: see Supplementary Figure S1 for a list of the oligos used in this study and their sequences. One of the component oligos had been 5′ end-labeled (^32^P), prior to annealing to the other component oligos. Twenty pmol of the radiolabeled oligo was mixed with 66 pmol of each of the other oligos that make up the construct. The oligos were annealed in 1× TPRT buffer (10 mM Tris–HCl (pH 8.0), 5 mM MgCl_2_, 200 mM NaCl) for 2 min at 95°C, followed by 10 min at 65°C, 10 min at 37°C and at last 10 min at room temperature. The constructs were not further purified post annealing as the procedure of gel purification led to inadvertent formation of partial junctions and gave us less control over DNA concentration. Junctions that shared a common labeled oligo were equalized by radioactive DNA counts; otherwise, equal volumes annealed junctions were used in R2 reactions.

R2Bm protein expression and purification were carried out for wild-type R2 protein, endonuclease mutant (KPD/A or K/ARNKY) and reverse transcriptase mutant (YAD/YD) as previously published (11,22). Briefly, *E. coli* BL21 cells containing the R2 expression plasmid were grown in LB broth and induced with IPTG. An empty expression vector was used to generate the protein extract that served as the mock-protein (øprotein) negative control in the functional assays. The induced cells were pelleted by centrifugation, resuspended and gently lysed in a HEPES buffer containing lysozyme and triton X-100. The cellular DNA and debris were spun down, and the supernatant containing the R2Bm protein was purified over Talon resin (Clontech #635501). The R2Bm protein was eluted from the Talon resin column and stored in protein storage buffer containing 50 mM HEPES pH 7.5, 100 mM NaCl, 50% glycerol, 0.1% triton X-100, 0.1 mg/ml bovine serum albumin (BSA) and 2 mM dithiothreitol (DTT) and stored at −20°C. R2 protein was quantified by sodium dodecyl sulphate-polyacrylamide gel electrophoresis (SDS-PAGE) along with a BSA standard titration and stained by SYPRO Orange (Sigma #S5692) prior to addition of BSA to the R2 protein for storage. All quantitations were done using FIJI software analysis on digital photographs (38).

### DNA binding, DNA cleavage and DNA synthesis reactions

R2Bm protein and target DNA binding, DNA cleavage and DNA synthesis reactions were performed largely as previously reported (11). Reactions were 13 µl and contained 80 fmol of labeled substrate DNA, 10-fold excess cold competitor DNA (dIdC) by mass, and a dilution series of R2Bm protein, typically ≥420 - ≤0.40 fmol protein. Each DNA construct was tested for its ability to bind to purified R2Bm protein and to undergo DNA cleavage in the absence of RNA (i.e. in the absence of 5′ PBM RNA and 3′ PBM RNA). The reactions were analyzed by native 5% polyacrylamide gel electrophoresis (EMSA) to determine fraction bound and denaturing (8 M urea) 8% polyacrylamide gels to determine fraction cleaved. A+G ladders as well as ladders made from different sized DNA oligos were run alongside the reactions in the denaturing urea gels to aid in mapping cleavages. Oligos used to build the constructs were used to also make the end labeled DNA oligo ladder and the A+G ladder. Only reactions in the linear range on a bound versus cleaved graph were used in determining cleavability, and then only from 20% bound to about 95% bound window as quantitation is problematic below and above that range. An SSS assay was performed by the addition of dNTPs to the DNA cleavage reactions. All gels were dried, exposed to a phosphorimager screen and scanned using a phosphorimager (Molecular dynamics STORM 840). The resulting 16-bit TIFF images were linearly adjusted (levels command) so that the most intense bands were dark gray. Adjusted TIFF files were quantified using FIJI software (38). Gel images presented in the main figures were adjusted (levels command) to visualize the cleaved and/or synthesized products of interest.

## RESULTS

### First-strand cleavage and TPRT products are poor substrates for second-strand cleavage

R2Bm inserts into a specific site in the 28S rDNA. In previous studies, it was determined that the protein subunit bound to target sequences downstream of the insertion site likely provides the endonuclease involved in second-strand (i.e. top-strand) DNA cleavage (6,10,20). Second-strand cleavage, however, has always been difficult to achieve and study. Previously, second-strand cleavage has required a narrow range of 5′ PBM RNA, R2 protein and DNA ratios. The prior data indicated that first-strand DNA cleavage is probably required before the second-strand can be cleaved, that the downstream subunit must be bound to the DNA (which required 5′ PBM RNA) and that the 5′ PBM RNA must then be dissociated from the downstream subunit for second-strand cleavage to occur (20). *In vivo*, with a full-length R2 RNA, the process of TPRT would be expected to pull the 5′ PBM RNA from the downstream subunit, putting the downstream subunit into the ‘no RNA bound’ state and thus initiating second-strand DNA cleavage.

**Figure 2.**
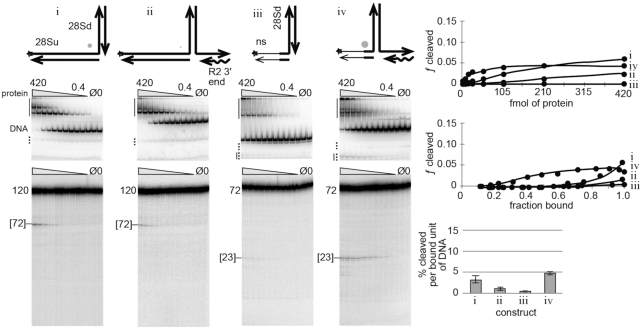
First-strand DNA cleavage and TPRT products are not good substrates for second-strand DNA cleavage. Several bottom/first-strand nicked linear DNAs (i and iii) and TPRT analogs (ii and iv) were tested for cleavability by the R2Bm protein. The 120 bp nicked 28S DNA (i) is diagrammatically bent at a 90° angle with the downstream (dwn) oriented toward the top of the page (i.e. the North arm). The TPRT product (ii) is similarly drawn; the TPRT (i.e. the East arm) arm is 25 bp. The star indicates that the DNA strand was 5′ end-labeled to track DNA binding and cleavage. In constructs iii and iv, the thin lines represent non-specific sequences; the left West arm was 25 bp and only the 5 bp nearest the second-strand cleavage site remained 28S DNA. Below each of the construct cartoons are the native (EMSA) gels and corresponding denaturing gels used to analyze DNA binding (EMSA) and DNA cleavage (denaturing) of the given DNA construct by R2Bm protein. DNA binding and cleavage reactions were 13 µl and contained 80 fmol of radiolabeled construct DNA and 420–0.4 fmol of R2Bm protein (gray triangle). All EMSA gels were quantified such that the bands above the full construct DNA in the mock purified protein (Ø) and no protein (0) control lanes were subtracted out of the bound signal in the experimental lanes. Solid vertical lines next to the EMSA gels represent areas of the gel where the bound DNA signal resides. The well, the smear and the gel migrating complexes were all counted as bound DNA. DNA bands located below unbound (free) DNA that increased with protein concentration were counted as bound DNA since these bands were released cleavage products. The released cleavage product co-migrated with partial junctions (dotted line) present in the control lanes. The control lane partial junction signal was subtracted from the experimental lane's co-migrating bound signal. The remaining partial junctions (dotted line) were counted as unbound DNA in the experimental lanes. The main band in the mock purified (Ø; protein purified from an empty expression vector) and the no protein (0) lanes is the location of the unbound junction DNA (DNA). Next to the denaturing gels is the size of the uncleaved radiolabeled oligo. The size and migration of the band resulting from second-strand cleavage is indicated by brackets on the denaturing gel. The DNA binding and DNA cleavage results are plotted on three graphs: (i) fraction (ƒ) cleaved as a function of protein concentration (fmol/reaction); (ii) fraction cleaved as a function of fraction bound for reactions where roughly 20–95% of the DNA was bound; and (iii) a bar graph reporting the average percentage cleaved products per bound unit of DNA (fraction bound) for reactions in the linear part of the second graph. The diameter of the gray dot next to each construct cartoon reflects the relative cleavability of the construct normalized to construct v in the next figure. See Supplementary Figure S2 for a graph of fraction bound as a function of protein concentration and for endonuclease mutant R2 protein controls.

In the first part of this study, the ability of the R2 protein to perform second-strand cleavage in the ‘no-RNA-bound’ state was investigated on products generated from the first two steps of the insertion reaction: first-strand DNA cleavage and TPRT (Figure 2). The product formed as a result of first-strand cleavage was made by annealing a 120 bp 28S derived target DNA containing 73 nt of 28S sequence upstream of the R2 insertion site and 47 nt of sequence downstream of the insertion site to two oligonucleotides complementary to the upstream and downstream segments of the 120 mer, respectively (10,20). In the diagram of the cleaved linear DNA in Figure 2 (construct i), the DNA has been bent 90° with the downstream 28S DNA ‘arm’ being oriented upward (‘North’) and the upstream 28S DNA arm remaining oriented to the left (‘West’). The TPRT product analog, construct ii, was similarly formed by annealing oligonucleotides. The TPRT analog included a 25 bp DNA/RNA heteroduplex arm derived from the 3′ end of the R2 element, positioned to the right (‘East’) in the diagram (3). The 120 nt ‘top’ (i.e. the sense) strand of the 28S gene was 5′ end-labeled with ^32^P to facilitate tracking of R2Bm protein induced DNA cleavage events (i.e. second-strand cleavage events). An electrophoretic mobility shift assay (EMSA) was used to measure the ability of the R2 protein to bind to each construct across a range of protein concentrations. Companion denaturing polyacrylamide gels were used to assay for second-strand DNA cleavage. The DNA binding and DNA cleavage data were quantified and are presented in several graphs: fraction cleaved as a function of protein concentration, fraction cleaved as a function of fraction bound, and a bar graph reporting the average percent cleaved per bound unit of DNA (derived from the linear portion of the fraction cleaved as a function of fraction bound graph).

Neither the first-strand cleavage product (construct i) nor the TPRT analog (construct ii) were good substrates for second-strand cleavage (Figure 2). DNA cleavage only occurred at or near protein excess, and even at these levels only a small percentage of the bound DNA was cleaved. This result is similar to the dynamics previously reported where it was not until the upstream protein binding site, located at −40 to −20 (see Figure 1B), was completely occupied did protein associate with the downstream DNA binding site resulting in cleavage of the sense strand of the 28S gene (6). And in the absence of RNA, as in the presence of 3′ RNA, the R2Bm binds to upstream DNA sequences (29).

In an effort to promote efficient second-strand cleavage, the upstream 28S DNA sequences of constructs i and ii were removed, forming constructs iii and iv. The upstream (West) arm of the two new constructs consisted of 25 bp of mostly nonspecific DNA; only the 5 bp prior to the second-strand cleavage site remained 28S sequence. The shortened first strand cleavage product (iii) failed to undergo second-strand cleavage. The TPRT product (iv) cleaved better than constructs i–iii and construct iv did not have the need for an excess of protein like i and ii. That said, only about 5% of the protein-bound constructs underwent second-strand DNA cleavage. Construct iv was still a poor substrate for second-strand cleavage.

### Specific open ‘4-way’ junctions are cleaved by R2 protein

**Figure 3.**
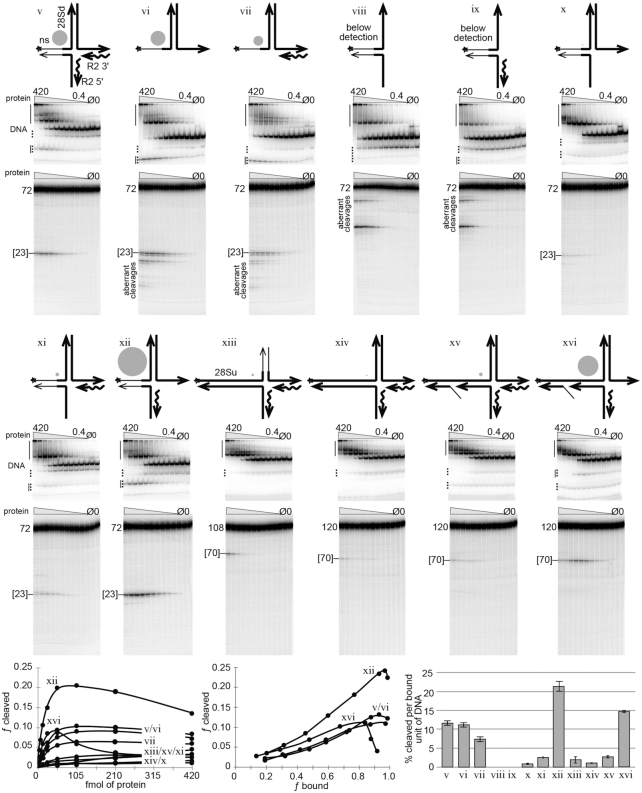
Specific open ‘4-way’ junctions are good substrates for second-strand DNA cleavage. Various R2Bm/28S derived junctions related to the open 4-way junction drawn in step 3 of the New Integration Model (Figure 1) were tested for DNA cleavage. Symbols, conventions, reactions, gels, analysis and graphs are as in Figure 2. The North arms of the constructs contain 47 bp of 28S downstream DNA, which is the same amount of downstream 28S DNA normally used in our linear target DNA (10,20). In construct xiii the 47 bp North arm was replaced with a 35 bp arm of mostly non-specific DNA. The 5 bp nearest the cleavage site, however, remained 28S DNA. The West arm of constructs v–xii were identical to constructs iii and iv (Figure 2), being 25 bp in length and containing mostly nonspecific DNA. The West arms of constructs xiii–xvi were 73 bp of upstream DNA and corresponds to the amount of upstream DNA normally used in our linear target DNA (6,29). East and South arms of all constructs are 25 bp. See Supplementary Figure S3 for mapping of DNA cleavages. See Supplementary Figure S4 for endonuclease mutant protein controls and for a graph of fraction bound as a function of protein concentration. See Supplementary Figure S5 for denaturing gels of specific EMSA bands.

In the second part of this study, open ‘4-way’ junctions that mimic the template switch hypothesized to occur at the close of TPRT were generated (see construct cartoons in Figure 3; see also *New Integration Model*, Figure 1C). A template switch is the association between the cDNA and the target DNA and the potential extension of the cDNA using the target DNA as a template. The 5′ end of the R2Bm mRNA is believed to contain rRNA sequence corresponding to the upstream target DNA (35,39–41). The reverse transcribed cDNA could then hybridize to the top strand of the target to form the 4-way junction.

**Figure 4.**
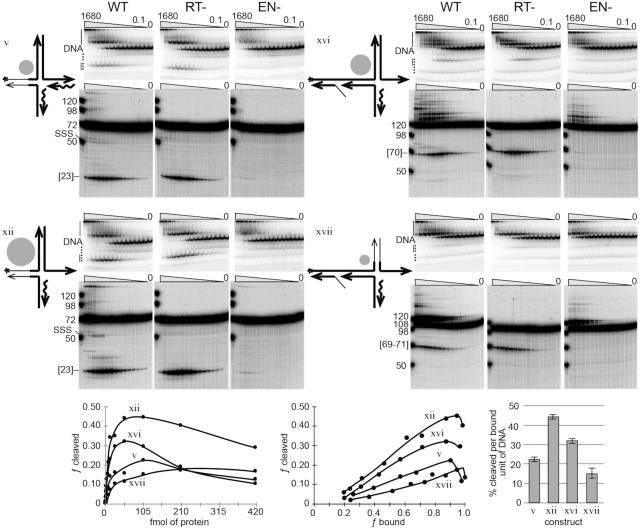
Second-strand DNA cleavage followed by second-strand DNA synthesis. Symbols, conventions, reactions, gels, analysis and graphs are as previous figures, except that dNTPs were added to the reactions. The reactions were carried out using wild-type R2 protein (WT), reverse transcriptase mutant protein (RT-) and endonuclease mutant protein (EN-). In addition, a broader protein titration range was used, 1680–0.1 fmol. The graphs include only 420–0.4 fmol range of the RT- cleavage data. A new construct, xvii, was included in this dataset in addition to constructs v, xii, xvi. The amount of SSS was not quantified as the signal is too low for reliable numbers. See Supplementary Figure S4 for a graph of fraction bound as a function of protein concentration for RT-dataset.

The open 4-way junctions generated in Figure 3 fell into three broad categories designed to analyze the sequence and structure requirements for precise and efficient DNA cleavage: (a) construct v and its derivatives vi–xii which have a short mostly non-specific upstream (West) DNA arm, (b) construct xiv and its derivatives xv–xvi, which have the full upstream and downstream 28S sequence (West and North arms) and (c) construct xiii, which has a medium length, mostly non-specific, downstream (North) DNA arm (see also the derivative construct xvii in Figure 4). The first and third groups of constructs limit the potential conformational space of the resulting protein–nucleic acid complexes due to the fact that known protein-binding-sequences are being removed. The second group of constructs retains the full upstream and downstream 28S sequences and the protein binding sites contained therein. All three groups of constructs retained 28S sequences proximal to the second strand DNA cleavage site (5 bp on either side, West and North arms). Multiple construct variations within each category were explored in order to more precisely define the DNA structure and sequence parameters required for second-strand cleavage. An R2 protein titration series was run on each labeled construct depicted in Figure 3. The labeled strand is marked with an asterisk (*) in the construct cartoons. The reactions were analyzed on native (EMSA) and denaturing polyacrylamide gels as in Figure 2. The gels for each construct are shown below the corresponding construct cartoon. The data that led to the mapping of the R2 cleavages, as well an endonuclease deficient R2 protein control for each construct, are located in the supplementary material (Supplementary Figures S3 and 4, respectively).

There are several parameters to consider in determining cleavability: (i) the amount of protein required to bind to the DNA, (ii) the amount of cleavage per protein-bound unit of DNA and (iii) the precision of the DNA cleavage. The second and third parameters were the most useful ones for comparing the cleavability between constructs. The first parameter was less informative because protein binding sites were being strategically removed and because of inherent issues with DNA quantitation when making the constructs and inherent issues with pipetting small volumes and protein (stored in glycerol) accurately.

Construct v was picked as a starting point in the analysis as construct v is the template-jump version of construct iv. The template-jump portion of construct v is the R2 5′ end (South) arm covalently attached to, and base paired with, the West arm. The South arm consists of a 25 bp cDNA/RNA duplex originating from the 5′ end of the R2 element RNA that would have been generated by TPRT. Construct v is a substantially better substrate for second strand cleavage than construct iv. Construct v cleaved about 11% of the protein-bound substrate. Interestingly, construct vi, which consisted only of the intact duplexed North arm and single stranded West and East arms (no South arm) cleaved just as well as construct v. The cleavage, however, was less precise. Only cleavages within few bases of the canonical R2Bm cleavage site were counted as second-strand cleavage for all constructs. Aberrant cleavages are marked as such on the denaturing gels and were not counted. Construct vii was structurally identical to construct vi, except it retained the original East arm heteroduplex. Construct vii, like construct vi resulted in imprecise cleavage at the R2 site and additional aberrant cleavages upstream of the R2 site on the single stranded West arm. A gray circle next to each construct cartoon represents the relative cleavability of the construct when normalized to construct v.

Both constructs viii and ix lack a duplexed North arm, resulting in aberrant cleavages at single stranded North arm and none at the R2 cleavage site. Because of the lack of cleavage at the R2 site, constructs viii and ix are noted in the figure as lacking detectable DNA cleavage.

Constructs x–xii test the result of having single-stranded East and/or South arms. The best substrate in terms of precision of cleavage and cleavage per bound unit of DNA was construct xii. Indeed construct xii was the best substrate out of the v-xii group of constructs, even better that construct v. The cleavage observed for constructs v and xii was primarily due to the R2 protein acting on the full construct and not on the present, but minor, partial junctions (dotted line on EMSAs); otherwise, constructs vi–ix, themselves being partial junctions of construct v, would have been cleaved better than they were. It is unfortunate that the (labeled) cleaved product in constructs v–vii and xii migrates at or very near a naturally occurring partial junction (solid line next to dotted line).

Construct xiii switched the non-specific DNA from the West arm to the North arm. The North arm was made only 35 bp long and retained 5 bp of 28S DNA located near the second-strand cleavage site. Construct xiv returns both North and West arms to the 28S derived DNA sequence containing the full R2 integration site. Both constructs xiii and xiv struggled to be cleaved. Construct xii showed dynamics similar to constructs i and ii (Figure 2), indicating that the protein is binding to the West arm and not the North arm at the lower protein concentrations. For complex iv, this dynamic was less so.

Constructs xv and xvi are direct analogs to the integration intermediate presented in step 3 of the *New Integration Model* (Figure 1). The West arm of these two constructs contained a ‘gap and a flap’ as a result of the of the template jump displacing the original DNA strand. The recombined cDNA/target DNA duplex portion of the West arm was 27 bp so as to match the amount of target sequence retained in the R2Bm transcript after processing by the R2 ribozyme (35). The 27 bp template jump/recombination places the gap and flap well into the upstream binding site (DNase footprint) for the R2Bm protein (29,35). The bifurcation of the West arm is thought to impart flexibility to the arm. Construct xv was not very cleavable, presumably because it had a heteroduplexed East arm. Construct xvi, however, with its single-stranded East arm cleaved well (15% cleavage per bound unit of DNA), second only to construct xii, which also happened to have a single stranded East arm. For example, a single stranded TPRT (East) arm would be expected to occur upon removal of the RNA from the cDNA/RNA heteroduplex by cellular RNase H activity. DNA cleavage decreased sooner on construct xvi than it did on either constructs v or xii in this data set (Figure 3). The early drop in cleavage for construct xvi is less pronounced in the data set presented in Figure 4 which was designed to test for SSS (see next section of the paper). The reverse transcriptase mutant (RT-) protein used in Figure 4 yields the same type of cleavage information as the wild-type protein (WT) used in Figure 3. Not only was the early drop in cleavage not as pronounced for construct xvi in Figure 4, but also the amount of cleavage per bound unit of DNA was nearly double for each of the constructs (v, xii, xvi) tested in Figure 4. The R2 protein used in Figure 4 was more active because it was fresher (1-day-old) than that used in Figures 2 and 3 which were age matched to 7 days old. DNA binding is long lived, but the amount of DNA cleavage per bound unit is age dependent. We also made a minor adjustment to the pH of our protein purification buffers. Datasets 2 and 3 were purified at pH 8, while the dataset in Figure 4 was returned to our traditional pH 7.5. The relative cleavage (gray dots) between constructs remained constant. Figure 4 also introduces a final construct. construct xvii. Construct xvii was similar to xvi with respect to the bifurcated West arm and single stranded East arm, but differs in that xvii had the non-specific North arm that construct xiii had. This construct surprisingly cleaved but was less precise; it cleaved the bases before and after (70–72) the cleavage site in addition to the canonical cleavage site at 71. Counting the cluster of cleavages as proper cleavage, the cleavability of construct xvii was significantly lower than that of construct xvi, but it otherwise had a similar fraction cleavage as a function of fraction bound profile to the other good substrates.

The relative ranking of the best substrates for second-strand DNA cleavage, normalized to the cleavability of construct v, was xii > xvi > v ≥ vi > vii ≥ xvii. Substrates with a single-stranded East arm were more cleavable by the R2 protein than substrates with a duplexed east arm. The data further indicated that the template-jump-derived West arm must be within a fairly narrow window of stability and that being too stable and rigid (xiii, xiv) seems to be inhibitory. Too low of a melting temperature leads to dissociation and concomitant loss of cleavage fidelity if the area remains single stranded (constructs vi,vii) or loss of cleavage if the structure returns to a TPRT-like 3-way junction (constructs ii, iv). A duplexed South arm, as opposed to a single-stranded South arm is required (e.g. compare x–xii). The bifurcated West arm appears to reduce/inhibit protein binding to only the upstream-28S R2-binding-site, although additional experiments will be needed to confirm, and DNA cleavage appears to be strongly associated with the North arm (i.e. downstream 28S DNA sequences, e.g. construct vi).

Protein–DNA complexes of constructs capable of being cleaved at the second-strand cleavage site by R2 protein form stable complexes that migrate within the EMSA gel, as opposed to being stuck in the well. Upon cleavage, the 4-way junction is resolved into two linear DNAs: one DNA containing the downstream (North) and R2 3′ (East) arms and one DNA containing the ‘upstream’ (West) and R2 5′ (South) arms. The West plus South DNA appears to be largely released by the R2 protein after cleavage, at least in the case of constructs iv–xii (see the lower solid vertical line in the EMSAs; see also Supplementary Figure S5). Some cleaved products for construct xvi can be found in the upper shifted region in the EMSA gel (i.e. still bound by protein) (Supplementary Figure S5). It is the West plus South DNA cleavage product that is expected to be the primer-template for second-strand DNA synthesis; as such, we would not expect it to be released by R2 protein *in vivo*. The fate of the North plus East half of the junction was not tracked post DNA cleavage.

### Second-strand cleavage leads to second-strand synthesis in the presence of dNTPs

The third and final part of this study was to explore SSS. To test if second-strand cleavage could progress to SSS, dNTPs were added to the DNA cleavage reaction. In addition, the reactions were carried out using wild-type R2 protein (WT), reverse transcriptase mutant protein (RT-) and endonuclease mutant protein (EN-). The WT protein cleaves and synthesizes DNA. The RT-protein cleaves but does not synthesize DNA. The data for the RT-protein is therefore analogous to the binding and cleavage reactions in Figure 3. The EN-protein does not cleave but still has an active reverse transcriptase. The mutation in the reverse transcriptase was YAD/YD and the endonuclease mutation was K/ARNKY (11,22). The EN-protein retained a low-level residual junction-cleavage-activity.

The best cleaving constructs, v, xii and xvi, along with the new construct, xvii, were used in reactions containing dNTPs (Figure 4) to test for SSS. The reactions were analyzed by denaturing polyacrylamide gel electrophoresis. The labeled strand of constructs v and xii was 72 nt uncleaved and 23 nt in length upon second-strand DNA cleavage. SSS, i.e. extension of the labeled strand post-DNA cleavage, would generate a 50 nt product when analyzed on a denaturing gel. The radiolabeled strand of construct xvi was 120 nt long uncleaved, 70 nt cleaved and 98 nt upon SSS. The labeled strand of construct xvii was 108 nt long uncleaved, 69–71 nt cleaved and 98 nt upon SSS. A larger range of R2 protein concentrations was used than in the previous figures. Second-strand DNA synthesis was observed on the denaturing gels for constructs v and xii at the higher end of the protein titration series. When the R2 RT gets to the end of the template, it adds on several untemplated nucleotides (42). The signal above the full-length oligo on the denaturing gels is the result of the original full-length oligo being extended by the R2 protein. The R2 protein can take almost any 3′ end and extend it, given a template in *cis* or in *trans* (42,43). The reason why full-length SSS was only prominent for constructs v and xii under conditions of protein excess was because the synthesis appears to be occurring primarily on the released primer template (lower vertical line on the EMSA) generated by second-strand cleavage and released from the protein/DNA complex. *In vivo*, it is not expected that the cleaved product would be released.

Partial SSS products were also detected, particularly in the case of construct xii. Several strong stops exist above the second-strand cleavage signal. These strong stops appear to occur as a direct result of synthesis being primed off the 3′-OH of the second-strand cleavage event; they are not present in either the RT- or the EN- datasets. The same stoppages were observed when construct xvi was used, indicating that priming of second strand synthesis also occurs on construct xvi. The strong stops may be the result of a structural constraint or required protein-DNA conformation change to switch from priming to elongation. The presence of the strong synthesis stops tracks strongly with the DNA cleavage profile.

## DISCUSSION

### A new model for R2Bm integration

The deeper understanding of the second half of the insertion reaction for R2Bm derived from the above experiments has allowed for an improved R2Bm integration model to be put forth (Figure 1C). The first half of the integration reaction is identical to steps 1 and 2 in the old (‘current’) integration model. After TPRT, however, the new integration model proposes a template jump or recombination event from the 5′ end of the R2 RNA to the top-strand of the 28S rDNA, upstream of the R2 insertion site, forming a 4-way junction (Figure 1C, step 3). It is this step that, to date, has not been shown to occur *in vitro* and may require host factors to form. An association of the cDNA to the upstream target DNA is consistent; however, with previous data, and the 4-way junction intermediate leads to a simple unified mechanism for 5′ junction formation and completion of integration.

Indeed, the new integration model makes sense of earlier *in vivo* experiments in which the ‘upstream’ ribosomal RNA sequence attached to the 5′ end of the R2Bm element RNA had been noted as a requirement for full-length element insertion (40,41). Studies have also determined that the R2 RNA is co-transcribed with ribosomal RNAs as part of the same large transcript (35,44). The R2 RNA is then processed from the bulk of the ribosomal RNA by a hepatitis delta virus (HDV)-like ribozyme found near the 5′ end of the R2 RNA (35,44). For a number of R2 elements, the processed R2 RNA retains some ribosomal RNA on the 5′ end, 27 nt of ribosomal RNA in the case of R2Bm (35). For elements that retain this much of the ribosomal RNA, the ‘template jump’ may be more of a strand invasion or recombination event than an actual template jump (40,41). For other R2 elements, however, the ribozyme leaves no ribosomal sequence on the processed R2 RNA (e.g. *Drosophila simulans* R2), and a template jump, as diagrammed in Figure 1C step 3 (of the new integration model), is envisioned to occur (16,35,39,43). The RT of both APE LINEs and RLE LINEs has been shown to have the ability to jump from the end of one template to the beginning of another without any homology (43). Template jumps have long been hypothesized to be involved in 5′ junction formation for both types of elements (16,35,39,43). In addition to template jumping, the reverse transcriptase of LINEs is able to use both DNA and RNA as templates during DNA synthesis and to displace a duplexed strand while polymerizing (16).

Recently, the R2 RLE’s reported similarity to Archaeal Holliday junction resolvases raised the question as to whether R2 binds and cleaves branched DNAs during integration (11,37). It turns out that the binding and cleavage of branched DNA is fundamental to the integration process itself. However, despite the formation and resolution of a ‘Holliday junction-like’ integration intermediate, with nearly symmetrical DNA cleavages, the R2 protein is not a Holliday junction resolvase. In fact, the cleavages are separated in time and arise via an activity much closer to that of a monomeric, single-stranded, DNA-endonuclease activity. Second strand cleavage appears to be the result of the endonuclease, and/or the R2 protein, associating with a double-stranded region and cleaving a nearby single-stranded region. This activity is exemplified by the cleavage data for constructs vi and vii in Figure 3. The other constructs that cleaved well, presumably, have a single stranded attribute to the cleavage site. Indeed, the cleavage site migrated between constructs as if in response to small local changes to the single-strandedness in the cleavage region (Figures 2–4; Supplementary Figure S6).

Similarly, there are good reasons to believe that first-strand cleavage and second-strand DNA cleavages are, at a fundamental level, identical with respect to how they arise since both instances are brought about by the same RLE. Indeed, DNase footprints of R2 protein bound to target linear DNA, prior to first-strand cleavage, show R2 protein induced DNase hypersensitive sites near the R2 cleavage/insertion site: local unwinding of a double helix would lead to DNase hypesensitive sites (6,29). Thus first-strand DNA cleavage may also be the result the endonuclease associating with a double stranded region and cutting a nearby single-stranded region.

Further, it is not known which part of the R2 protein binds the 4-way DNA junction. It may or may not be the endonuclease (45). It remains to be investigated whether the jump/recombination event precludes protein binding to the upstream (−40 bp to −20 bp) binding site, as our results suggest.

Cleavage of the 4-way junction generated a natural primer-template used for second-strand DNA synthesis. In our *in vitro* reactions, however, much of the primer-template is released after cleavage. As such, it remains an open question as to wether or not R2 provides this function *in vivo*. It is encouraging, however, that construct xvi yielded SSS products in the form of constrained synthesis. It is possible that the priming occurred on cleaved substrates still bound by protein.

#### One protein subunit or two?

Is the integration reaction performed by one protein subunit or two? It is an open and unresolved question. The two subunit model presented in Figure 1B and C (current integration model), still fits all the data. That said, the one subunit model also fits most, if not all, of the data. The new data has the R2 protein recognizing a sequential set of complicated branched DNA structure(s). Each arm of the branched structure(s) appear to have their own sequence and local structure requirements that must be met for integration to occur. Our new data intellectually fits well with a one subunit ‘rock and roll’ model. In the rock and roll model, the R2 protein is bound to the 3′ PBM RNA and thus binds to the upstream 28S DNA (West arm) on the linear DNA. Binding of the upstream R2 protein subunit to the target DNA induces local unwinding at the R2 site. The endonuclease of the upstream bound R2 protein ‘rocks’ into place and cleaves the single-stranded R2 site. The reverse transcriptase of the upstream bound R2 protein is rocked into place and begins TPRT. The initial stages of TPRT removes the 3′ PBM RNA from the protein (due to heteroduplex formation). The 5′ PBM RNA associates with the R2 protein and the protein adopts the downstream binding conformation; the protein ‘rolls’ to the North arm while also making potential contacts with the TPRT (East) and West arms. TPRT finishes, removing the 5′ PBM RNA from the protein (due to heteroduplex formation). The R2 protein is now in the minus RNA state. The template jump occurs to form the open 4-way junction the R2 protein rolls to bind the 4-way junction as described in the ‘Results’ section. The endonuclease rocks into place and cleaves the open 4-way junction. The reverse transcriptase is then rocked into place to perform second-strand DNA synthesis. More experiments are needed to determine which model, one or two subunit, is correct and to more fully understand the integration reaction.

### Extrapolating the R2 model to LINEs with different cleavage staggers

**Figure 5.**
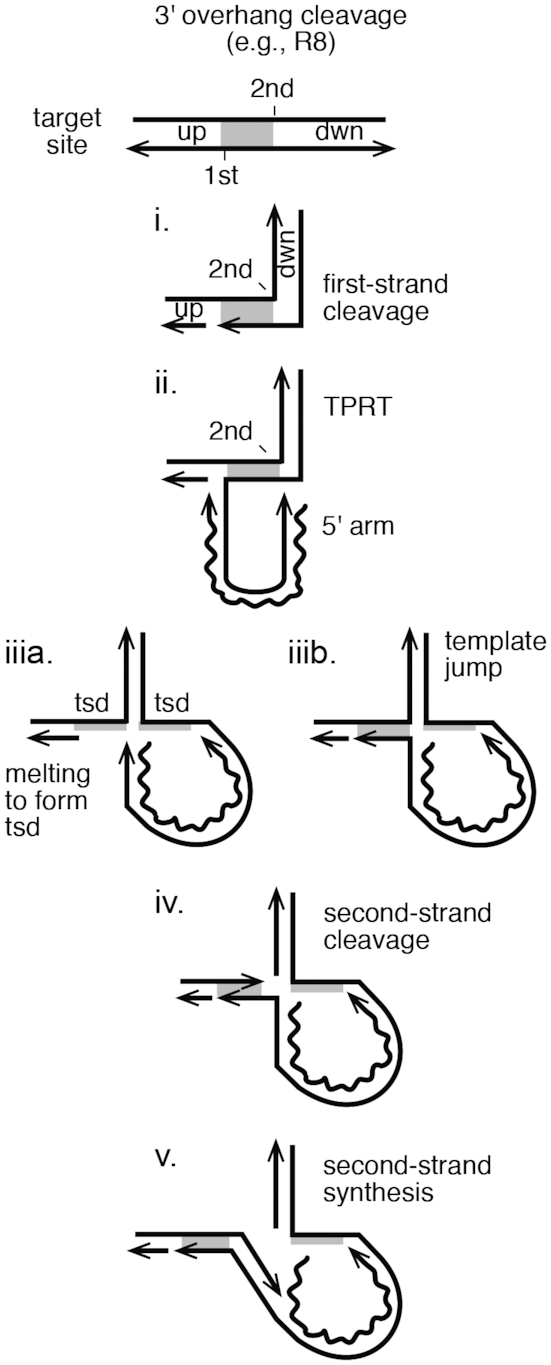
Extrapolating the New Integration Model to RLE LINES that generate target site duplications. A target site is diagrammed with the first- and second-strand DNA cleavages staggered such that a target site duplication (tsd) would occur upon element insertion. The steps are as in R2 integration, except that the template jump displaces/melts the DNA between the two cleavages to generate the open 4-way junction and the tsd upon DNA synthesis.

The position of the second-strand DNA cleavage site relative to the first-strand cleavage site is variable across species, and even more so across the R2 clade. The stagger of the first and second DNA cleavage events in R2Bm is a small 5′ overhang of 2 bp that leads to 2 bp target site deletion upon insertion of the element. In *Drosophila melanogaster* the R2 endonuclease produces blunt cleavages (39). Other R2 elements produce small 3′ overhangs (26). The 3′ prime overhanging staggered cuts produce target site duplications instead of deletions. The model presented in Figure 1 works equally well for elements with any form of small staggers. The model easily can be adapted for elements that generate larger target site duplications. The R8 element in *Hydra magnipapillata* generates a 9 bp target site duplication upon insertion (46). The R4 element generates a 13 bp target site duplication (46). The model for elements like R8 and R4 is presented in Figure 5. The difference between the model in Figure 1, where the cleavage stagger is small, and that proposed for R8 is that a local melting or displacement of the region between the cleavage sites is hypothesized to occur along with the template switch, generating the 4-way junction.

APE LINEs also tend to produce a 3′ overhanging stagger in the range of 10–20 bp. It remains to be determined if APE LINEs use a 4-way junction structure to drive second-strand DNA cleavage and synthesis. Bioinformatic analysis of 5′ junctions of full-length L1 and Alu elements is suggestive of template jumping to the upstream target sequence and that DNA repair might be an alternative path to 5′ junction formation for abortive insertion events (1,15,17,47,48). Twin priming in L1 might be a related, albeit aberrant, phenomenon to SSS (49). An association between the cDNA and the upstream target DNA has been hypothesized for some R1 elements (39). Ribosomal sequences are also important for element–RNA/target–DNA interactions during first-strand synthesis for R1Bm as well as several other site-specific LINEs, but they do not appear to be as important for R2Bm (26,50,51).

## Supplementary Material

gkz673_Supplemental_File
