# Supplementary material for: Completion of LINE integration involves an open ‘4-way’ branched DNA intermediate
Source: Nucleic Acids Res. 2019 Aug 8;47(16):8708–19. doi: 10.1093/nar/gkz673 (PMC6895275; doi:10.1093/nar/gkz673)
Supplement: gkz673_Supplemental_File [file gkz673_supplemental_file.pdf]

Supplemental S1: Oligonucleotides used in the study.

Name	Oligonucleotide Sequence (5'---3')
**NS / 28Sd 25bp**	TCCAGAAGCTTCCGGTAGCTTAAGGTAGCCAAATGCCTCGTCATCTAATT
**Comp 28Sd 25bp / NS**	AATTAGATGACGAGGCATTTGGCTACCTTAAGCTACCGGAAGCTTCTGGA
**Pre-cleaved (1) Comp 28Sd 25bp; (2) NS**	(1) AATTAGATGACGAGGCATTTGGCTA<br>(2) CCTTAAGCTACCGGAAGCTTCTGGA
**R2 3' DNA 25bp / NS**	TGGCATGATGATCCGGCGATGAAAACCTTAAGCTACCGGAAGCTTCTGGA
**R2 3' DNA 25bp / R2 5' DNA 25bp**	TGGCATGATGATCCGGCGATGAAAAGGGGCGATACGCATAATTTTAATTT
**R2 3' DNA 25bp**	TGGCATGATGATCCGGCGATGAAAA
**R2 3' RNA 25bp**	UGGCAUGAUGAUCCGGCGAUGAAAA
**R2 5' DNA 25bp**	GGGGCGATACGCATAATTTTAATTT
**R2 5' RNA 25bp**	GGGGCGAUACGCAUAAUUUUAAUUU
**Comp 28Sd 25bp / R2 3' DNA 25bp**	AATTAGATGACGAGGCATTTGGCTATGGCATGATGATCCGGCGATGAAAA
**Comp 28Sd 25bp / Comp R2 3' DNA 25bp**	AATTAGATGACGAGGCATTTGGCTATTTTCATCGCCGGATCATCATGCCA
**Comp R2 3' RNA 25bp / NS**	TTTTCATCGCCGGATCATCATGCCACCTTAAGCTACCGGAAGCTTCTGGA
**Comp R2 5' RNA 25bp / NS**	AAATTAAAATTATGCGTATCGCCCCCCTTAAGCTACCGGAAGCTTCTGGA
**NS / 28Sd 47bp**	TCCAGAAGCTTCCGGTAGCTTAAGGTAGCCAAATGCCTCGTCATCTAATTAGTGACGCGCATGAATGGATTA
**Comp 28Sd 47bp / Comp R2 3' RNA 25bp**	TAATCCATTCATGCGCGTCACTAATTAGATGACGAGGCATTTGGCTATTTTCATCGCCGGATCATCATGCCA
**28Su 73bp / NS**	GCTCTGAATGTCAACGTGAAGAAATTCAAGCAAGCGCGGGTAAACGGCGGGAGTAACTATGACTCTCTTAAGGTAGG<br>GTCCAGAAGCTTCCGGTAGCAGCGAGAGCGG
**Comp NS / Comp R2 3' RNA 25bp**	CCGCTCTCGCTGCTACCGGAAGCTTCTGGACCCTATTTTCATCGCCGGATCATCATGCCA
**Comp R2 5' RNA 25bp / Comp 28Su 73bp**	AAATTAAAATTATGCGTATCGCCCCCCTTAAGAGAGTCATAGTTACTCCCGCCGTTTACCCGCGCTTGCTTGAATTTCT<br>TCACGTTGACATTCAGAGC
**28Su 73bp / 28Sd 47bp**	GCTCTGAATGTCAACGTGAAGAAATTCAAGCAAGCGCGGGTAAACGGCGGGAGTAACTATGACTCTCTTAAGGTAGCC<br>AAATGCCTCGTCATCTAATTAGTGACGCGCATGAATGGATTA
**Comp R2 5' RNA 25bp / Comp 28Su 27bp**	AAATTAAAATTATGCGTATCGCCCCCCTTAAGAGAGTCATAGTTACTCCCG
**Flap Comp 28Su**	ATATATGTTTACCCGCGCTTGCTTGAATTTCTTCACGTTGACATTCAGAGC

Supplemental S2

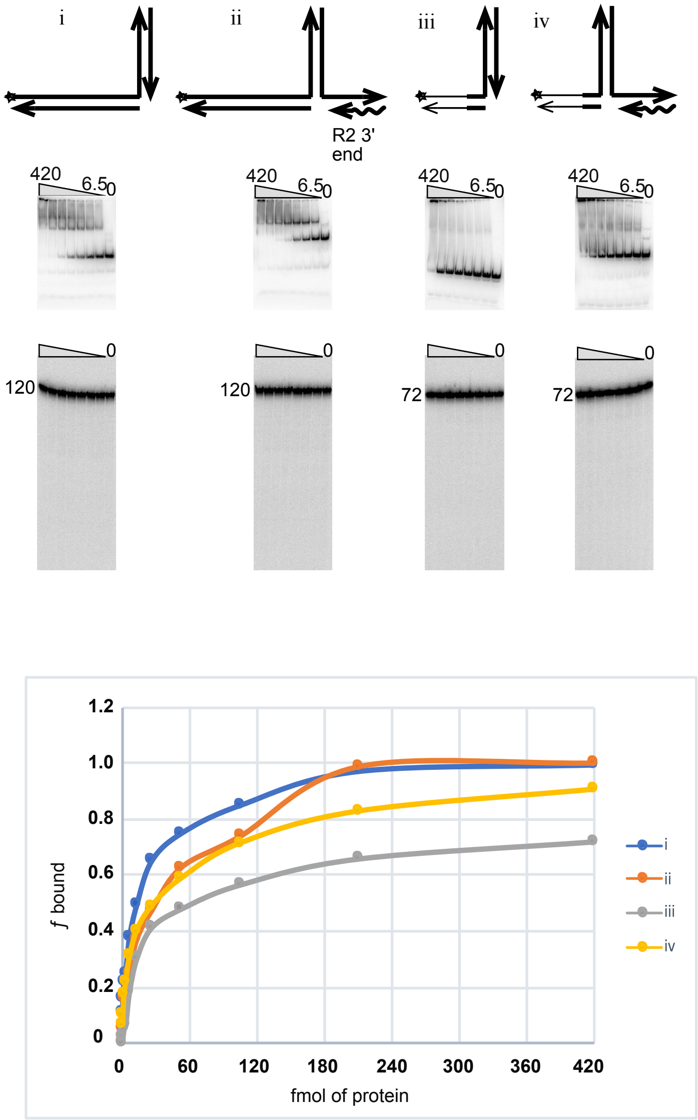

Supplemental S2: Related to Figure 2. EMSA gels of R2Bm protein acting on constructs i-iv across a range of endonuclease mutant (EN-) R2Bm protein concentrations (420-6.5 fmol). A cartoon of each construct is presented. Under the construct cartoon is the native gel (EMSA) analysis and corresponding denaturing gel for each construct. The exposure of the gels in this figure was linearly adjusted such that the bands, if any, would readily be visible. A graph of average DNA bound (*ƒ* bound) as a function of protein concentration (fmol/reaction) is shown for constructs i-iv.

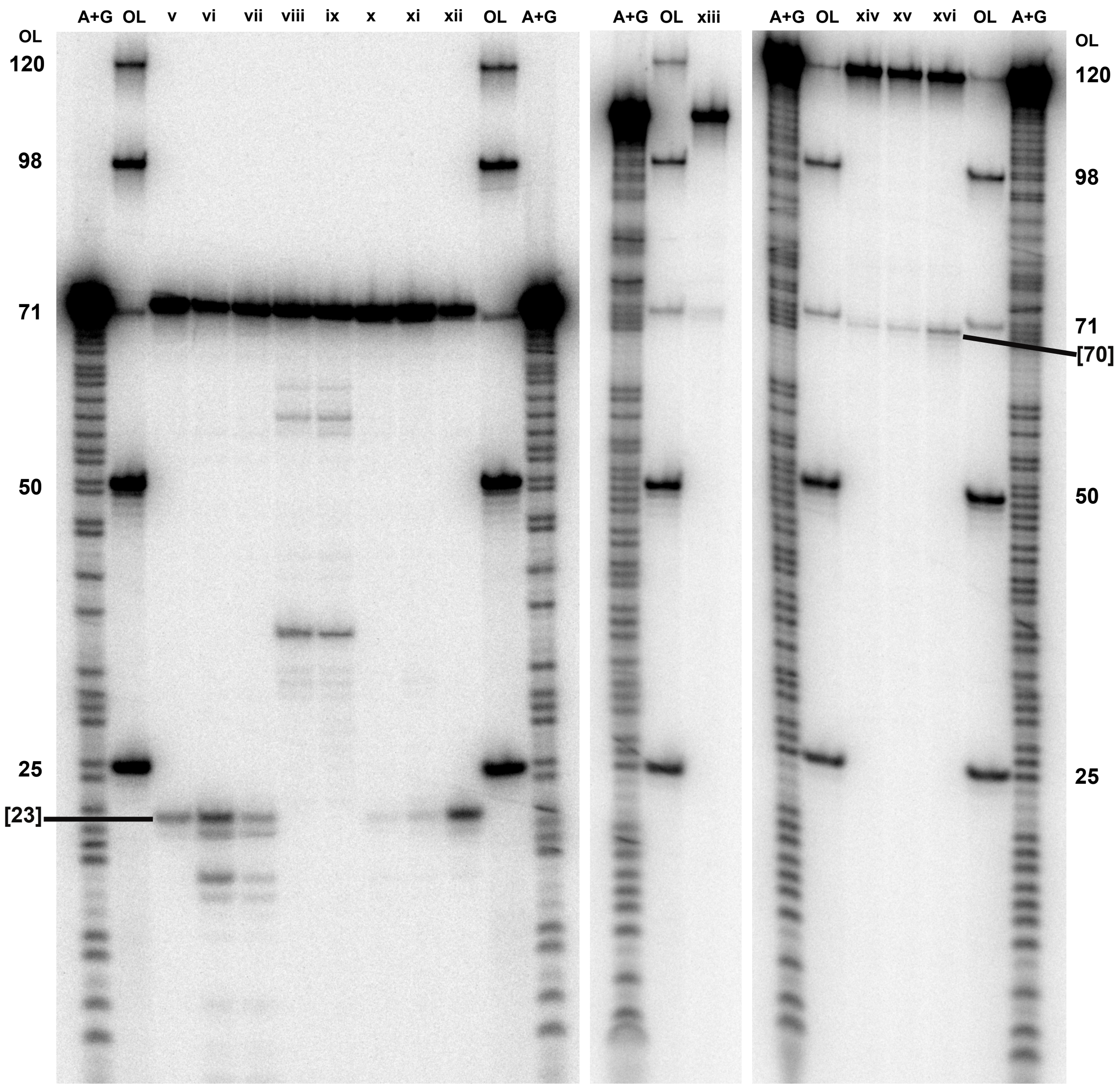

Supplemental S3: Related to Figure 3. Urea-denaturing 8% polyaclylamide gel showing mapping of second-strand DNA cleavages for constructs v-xvi. The *in vitro* reactions were carried out using wild type R2Bm protein (WT) for each construct listed at the top of the gel. A+G ladder as well as ladders made from different sized DNA oligos (as indicated on the figure) were run alongside the reactions to aid in mapping of second-strand DNA cleavage. The exposure of the gels in this figure was linearly adjusted such that bands resulting from DNA cleavage were readily visible.

Supplemental S4

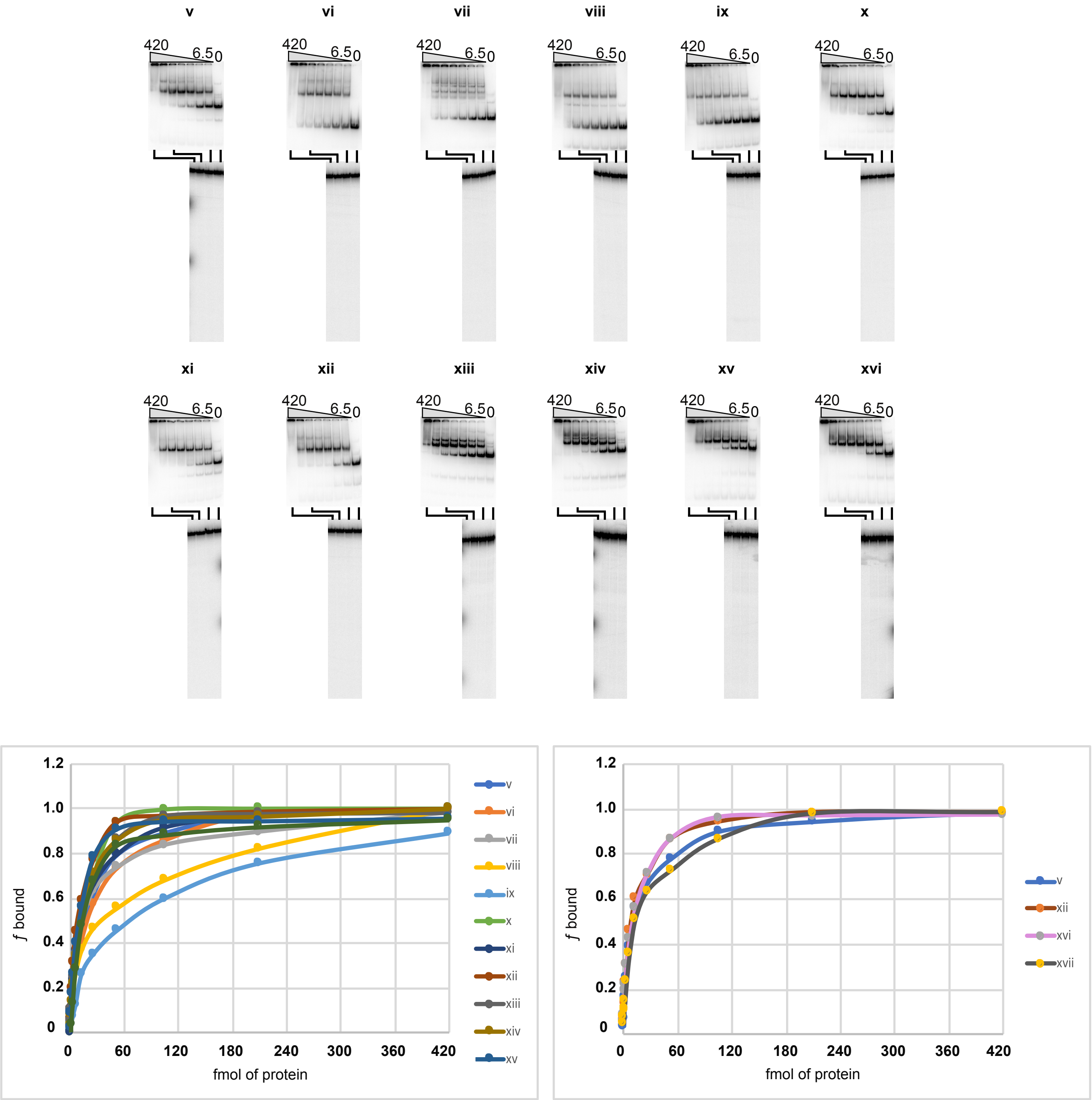

Supplemental S4: Related to Figure 3. EMSA gels and urea-denaturing 8% polyacrylamide gels showing data for endonuclease mutant protein (EN-) acting on constructs v-xvi across a range protein concentration (420-6.5 fmol). Native gel (EMSA) analysis is presented. Under each native gel (EMSA) are the corresponding denaturing gel for each construct. Each lane on denaturing gel represents a specific protein concentration of the titration series on the corresponding EMSA gel, as indicated by straight lines. The exposure of the gels in this figure was linearly adjusted such that the bands, if any, would readily be visible. A graph of average DNA bound (ƒ bound) as a function of protein concentration (fmol/reaction) are shown for constructs v-xii. Also, a graph of average DNA bound (ƒ bound) as a function of protein concentration (fmol/reaction) are shown for constructs v-xviii (Related to Figure 4).

Supplemental S5

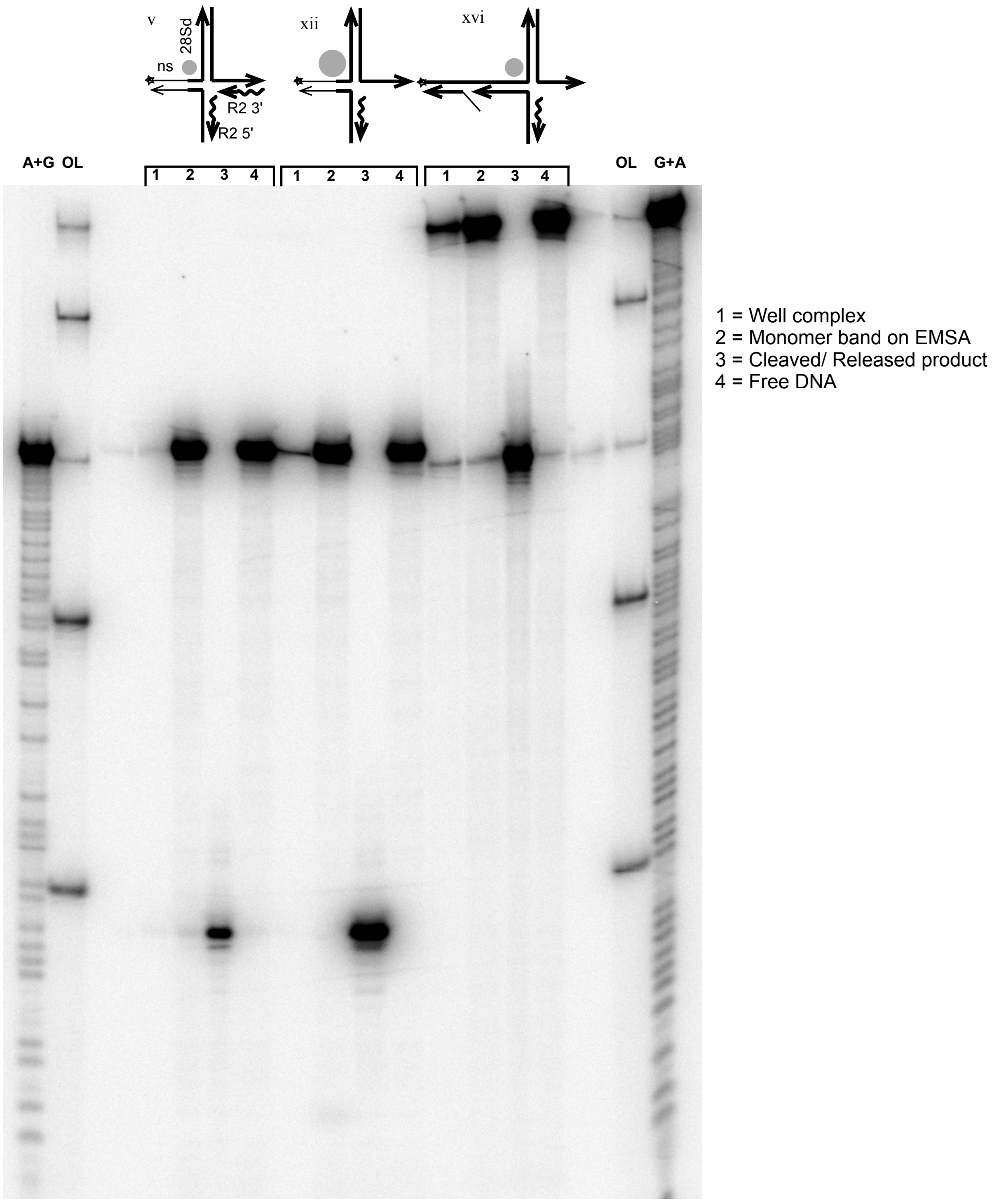

Supplemental S5: Related to Figure 3. Urea-denaturing 8% polyaclylamide gel showing mapping of cleaved and released products for constructs i, viii and xii. A cartoon of each construct is shown above each corresponding set [1-4; described each next to denaturing gel]. The *in vitro* reactions were carried out using wild type R2Bm protein (WT) for each construct. A+G ladder as well as ladders made from different sized DNA oligos (as indicated on the figure) were run alongside the reactions to aid in mapping cleaved and released products. The exposure of the gels in this figure was linearly adjusted such that bands resulting from DNA cleavage and product released were readily visible.

Supplemental S6

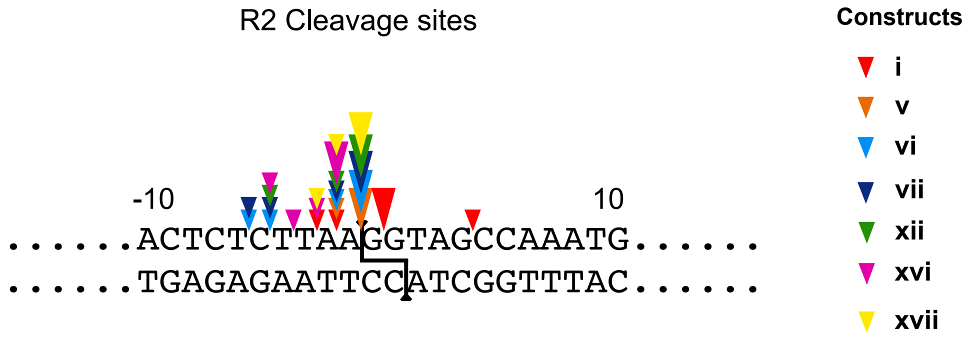

Supplemental S6: Diagram of the second-strand cleavages on the 28S target. Colored triangles indicate second-strand cleavage for designated constructs. Nucleotide positions are numbered with respect to the central dyad. The canonical cleavage site are indicated by black arrows. Each construct shows a major cleavage indicated by a "larger" triangle and other cleavage indicated by "smaller" triangles. The cleavages for construct i were determined by looking at the denaturing gel in Figure 5 from 2004 Christensen and Eickbush paper (Christensen and Eickbush, 2004).
